# Evaluation of the efficacy and safety of *Escherichia coli*-derived recombinant human bone morphogenetic protein-2 in transforaminal lumbar interbody fusion to treat degenerative spinal disease: a protocol of prospective, randomized controlled, assessor-blinded, open-label, multicenter trial

**DOI:** 10.1186/s13018-022-03289-w

**Published:** 2022-08-31

**Authors:** Jun-Young Choi, Hyun-Jin Park, Sang-Min Park, Chang-Nam Kang, Kwang-Sup Song

**Affiliations:** 1 Spine Center and Department of Orthopaedic Surgery, Seoul National University College of Medicine and Seoul National University Bundang Hospital, 82, Gumi-ro 173 Beon-gil, Bundang-gu, Seongnam-si, Gyeonggi-do 13620, Seongnam, Republic of Korea; 2 Department of Orthopedic Surgery, Spine Center, Kangnam Sacred Heart Hospital, Hallym University College of Medicine, Seoul, Korea; 3 Department of Orthopedic Surgery, Hanyang University College of Medicine, Seoul, Republic of Korea; 4 Department of Orthopedic Surgery, Chung-Ang University College of Medicine, Seoul, Republic of Korea

**Keywords:** Degenerative lumbar disorders, Recombinant human bone morphogenetic protein-2, Transforaminal lumbar interbody fusion, Randomized controlled trial

## Abstract

**Background:**

Recombinant human bone morphogenetic protein-2 (rhBMP-2) has been widely used as an alternative bone graft in spine fusion surgery. However, clinical outcome such as effects and complications has not yet been revealed for transforaminal lumbar interbody fusion (TLIF). Although previous studies have reported some results, the evidence is weak. Therefore, the purpose of this trial is to evaluate the effectiveness and safety of *Escherichia coli*-derived rhBMP-2 combined with hydroxyapatite (HA) in TLIF.

**Methods:**

This trial is designed as a prospective, assessor-blinded, open-label, multicenter, randomized controlled study. Participants will be recruited from six tertiary teaching hospitals. All randomized participants will be undergoing one- or two-level TLIF with rhBMP-2 (77 participants) as the active experimental group or with an auto-iliac bone graft (77 participants) as the control group. The primary interbody fusion rate outcome will be evaluated using computed tomography (CT) 12 months after surgery. The secondary outcomes will be as follows: clinical outcomes (visual analog scale score, EuroQol-5-dimensions-5-level score, Oswestry Disability Index score, and some surgery-related variables) and adverse effects (radiculitis, heterotrophic ossification, endplate resorption, and osteolysis). Radiological outcomes will be evaluated using simple radiography or CT. All outcomes will be measured, collected, and evaluated before surgery and at 12, 24, and 52 weeks postoperatively.

**Discussion:**

This study will be the primary of its kind to evaluate the effectiveness and safety of *E. coli*-derived rhBMP-2 with HA in one- or two-level TLIF. It is designed to evaluate the equivalence of the results between rhBMP-2 with HA and auto-iliac bone graft using an appropriate sample size, assessor-blinded analyses, and prospective registration to avoid bias. This study will set up clear conclusions for using *E. coli*-derived rhBMP-2 with HA in TLIF.

*Trial registration*: This study protocol was registered at Korea Clinical Research Information Service (https://cris.nih.go.kr; number identifier: KCT0005610) on 19 November 2020. And protocol version is v1.1, January 2022.

## Background

After the discovery of bone morphogenetic proteins by Marshall Urist in 1965 [1], the frequency of osteoinductive growth factor use in spinal surgery has significantly increased [2–5]. The use of recombinant human bone morphogenetic protein-2 (rhBMP-2) during lumbar fusion surgery as an alternative to autogenous bone graft can minimize complications, such as pain at the donor site, and it has been confirmed that it has the same effect as autologous long bone during spinal fusion [6, 7]. When rhBMP-2 was used in anterior lumbar interbody fusion (ALIF) and lateral lumbar interbody fusion (LLIF), high fusion rates were observed, respectively (94.5% and 92%) [8, 9]. Furthermore, the union rate of the transforaminal lumbar interbody fusion (TLIF) treated with rhBMP-2 was 96.6%, which was higher than that of the non-rhBMP-2 group (92.5%) [10, 11]. Therefore, rhBMP-2 may be a good substitute for bone fusion, which could be used to reduce iliac bone graft donor site morbidity [6]. Despite its high osteoinductivity, rhBMP-2 is difficult to apply clinically because it is extracted from mammals and expensive. However, *Escherichia coli*-derived rhBMP-2 shows a better extraction rate than that of mammals, and high osteogenic activity and cost-effectiveness have been reported [12, 13].

There is still no clear evidence on the clinical and radiographic efficacy and safety of *Escherichia coli*-derived rhBMP-2 on TLIF surgery. Therefore, a randomized controlled trial (RCT) is considered necessary. Specifically, we will compare the safety of using *E. coli*-derived rhBMP-2 with hydroxyapatite (HA) or autogenous iliac bones in patients in need of TLIF to complement the limitations of previous studies and confirm whether the *E. coli*-derived rhBMP-2 with HA bone union rate is non-inferior to that of autogenous iliac bone, as well as its clinical efficacy and safety.

## Methods

### Ethics statements

The design and protocol of this trial were approved by the institutional review board (IRB) of our hospitals (IRB number: B-2008-630-003). Contrary to this trial plan, any changes that could affect future research data will be approved by the research ethics committee before conducting. Informed consent will be obtained from all participants. The results will be submitted to peer-reviewed publications.

### Trial design and participants

**Fig. 1:**
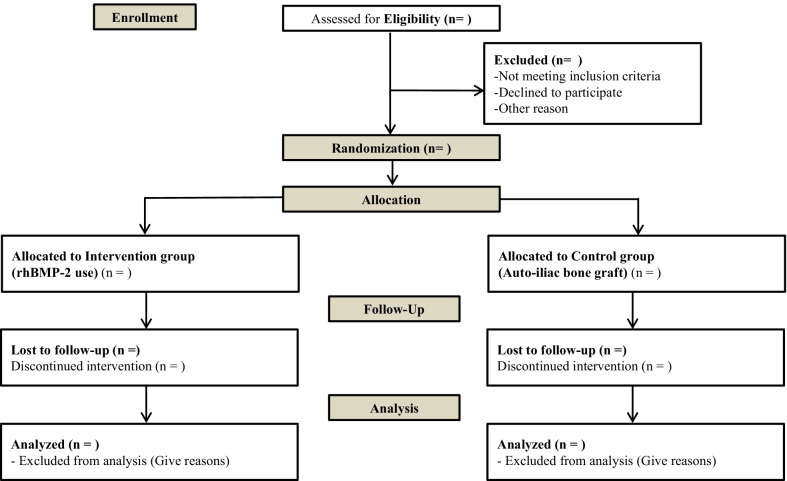
CONSORT study flow diagram of this study protocol

In this multicenter, assessor-blinded, open-label, prospective RCT, a total of 154 adults (age, 19–80 years) who will be undergoing TLIF for degenerative lumbar disease will be recruited from six tertiary teaching hospitals to evaluate the efficacy and safety of *E. coli*-derived rhBMP-2 combined with HA in TLIF. Participants will visit the hospital at 12, 24, and 52 weeks postoperatively, so we can evaluate their outcomes. Eligibility for enrollment is based on the following inclusion and exclusion criteria (Table 1, Fig. 1).

**Table 1:** Inclusion and exclusion criteria

Inclusion criteria
Age 19–80 years
Patients needing TLIF and posterior pedicle screw fixation at one or two levels between L1 and S1 because of one of the following degenerative lumbar diseases (including patients who do not respond despite 3 months of non-surgical treatment or who have persistent pain with a VAS score ≥ 4):
Lumbar spinal stenosis
Severe herniated intervertebral disk requiring wide laminectomy
Spondylolisthesis or spondylolysis
Recurrent herniated intervertebral disk
Patients able to understand and consent to the study
Patients willing to participate and comply with our proposed follow-up protocol

Exclusion criteria
History of lumbar fusion surgery at the same level
Pregnant, lactating, or childbearing-age patients who do not agree to maintain contraception during the clinical period
Patients with severe osteoporosis (lumbar T-score < − 3.0)
History of malignancy (but patients can participate if the disease has not been relapsed within the last 5 years after being cured)
Hypersensitivity to rhBMP-2, HA, or the PEEK cage
Patients with any of the following diseases that make it difficult to proceed with the protocol (e.g., psychological disorders, drug abuse, abnormal liver, kidney, heart, respiratory function, infectious disease, metabolic disease, ankylosing spondylitis, tumor, trauma, etc.)
Patients taking the parathyroid hormone or bisphosphonate for osteoporosis
Patients with autoimmune disease who need to take high-dose steroids for a long time
Patients who smoke > 20 cigarettes a day
Body mass index > 35 kg/m^2^
Patients who cannot stop taking antithrombotic drugs or anticoagulants before surgery and are expected to have bleeding during surgery
Severe diabetic mellitus patients with complications
A person whose life expectancy is judged to be short (terminal patient)
Patients unable to understand and consent to the protocol

*TLIF* transforaminal lumbar interbody fusion; *VAS* visual analog scale; *rhBMP-2* recombinant human bone morphogenetic protein-2; *HA* hydroxyapatite; *PEEK* polyetheretherketone

### Recruitment

Participants will be recruited if they are going to receive TLIF for lumbar degenerative disease at one or two levels in six hospitals. A blinded assessor will conduct the baseline screening to include participants. All participants will choose to participate in the trial after receiving an oral explanation by the evaluators and providing written informed consent of the same content. No monetary benefit will be given to the participants, and no added harm or adverse events are anticipated over traditional instrumented fusion surgery.

### Randomization

The included participants will be randomly assigned to two groups at a 1:1 ratio: an active rhBMP-2 group and a control auto-iliac bone group, following a computer-generated randomization list prepared by a researcher. This lists will be integrated into a web-based eCRF platform that is accessible to researchers who had qualified at this study. This process will be implemented independently for each of the six hospitals, and the surgeon will be notified of the results immediately before surgery.

Participants randomized to the active *E. coli*-derived rhBMP-2 group will be treated with *E. coli*-derived rhBMP-2 and HA during TLIF. The dose of *E. coli*-derived rhBMP-2 at each segment will be 0.5–1.0 mg, and the maximum dose for one participants will be 2.0 mg. Participants randomized to the control auto-iliac bone group will receive autogenous iliac bone from the posterior iliac crest.

### Interventions

- Active intervention: rhBMP-2 with HA and local lamina bone
- Control intervention: the auto-iliac bone graft and local lamina bone

The surgical methods of the active group using rhBMP-2 and the control group using auto-iliac bone graft will be similar except for the procedure of filling the cage in the intervertebral space. TLIF will be routinely performed. Following the posterolateral approach, decompression with foraminotomy and flavectomy will be performed. Total discectomy and endplate preparation will follow. In the active rhBMP-2 group, rhBMP-2 with HA and local lamina bone will be placed in the PEEK cage in the intervertebral body space. Novosis® (CGBio Inc., Gyeonggi-do, Korea), which consists of 0.5 g of HA and 0.5 mg of *E. coli*-derived rhBMP-2, will be used for each segment. In contrast, the control group will receive 5 to 10 cc of auto-iliac bone graft and local lamina bone for each segment. A PEEK cage will be inserted in the intervertebral body space. Percutaneous pedicle screw fixation will be performed at the involved lumbar level. Wound closure will be conducted after drainage.

### Outcome measures

#### Primary outcome

The primary outcome is the interbody union rate at 52 weeks after surgery. The status of the interbody union rate will be evaluated using the ‘bone bridge ratio.’ It will be assessed on coronal and sagittal CT images (1-mm interval cutting image). It is evaluated as the ratio of the bone bridge length to the total length of the upper and lower end plates of the segment. The study will be conducted by two orthopedic surgeons who do not participate in the study.

#### Secondary outcomes

##### Radiographic outcomes

Simple lumbar radiographs, which consist of anterior–posterior, lateral, lateral-flexion and lateral-extension views, will be used to confirm the overall condition of the participant's lumbar spine before surgery. During follow-up, simple radiographs will be obtained to assess for adverse events, such as heterotrophic ossification, cage subsidence, screw loosening, and adjacent segment disease.

##### Clinical outcomes

Patient-reported outcomes, which includes the Oswestry Disability Index (ODI) score for lumbar disabilities, EuroQol-5-dimensions-5-level (EQ-5D-5L) value for overall quality of life, and VAS pain scores, will be collected before surgery and AT 12, 24, and 52 weeks postoperatively. Safety will be assessed by evaluating adverse events and effects which is related to surgery. Adverse events and surgery-related effects will be made report to the appropriate institution. Laboratory tests for the rhBMP-2 antibody will be performed before surgery and at 12 weeks after surgery. These results and data will be compiled by a blinded assessor and enrolled using the eCRF system (Table 2).

**Table 2:** Evaluation schedule

Visit type	Screening	Operation	Follow-up		
Visit	1	2	3	4	5
Visit week	− 4 ~ 0 weeks	0 ~ 2 days	12 weeks	24 weeks	52 weeks
			± 5 weeks	± 8 weeks	± 8 weeks
Trial description and consent	○				
Demographic data^a^	○				
Physical examination	○				
Vital sign	○				
Laboratory test^b^	○				
Bone mineral density	○				
Sampling for rhBMP-2 antibodies^c^	○		○		
Inclusion/Exclusion criteria	○	○			
Randomization		○			
Operation		○			
Simple radiographs	○				
Computed tomography					○
ODI	○		○	○	○
EuroQol-5-dimensions	○		○	○	○
VAS	○		○	○	○
Union rate evaluation^d^					○
Concomitant drug		○	○	○	○
Adverse events		○	○	○	○

*rhBMP-2* recombinant human bone morphogenetic protein-2; *ODI* Oswestry Disability Index; and *VAS* visual analog scale

^a^Baseline patient characteristic, including past medical/surgical history, physical examination

^b^including CBC, chemistry, urinalysis, urine HCG test

^c^Test for BMP-2 antibody baseline. Applicable only to the rhBMP-2 control group after surgery

^d^Evaluation in simple radiographs and CT by 2 independent orthopedic specialists who do not participate in this study process

### Sample size

This clinical study will compare the interbody fusion rate between the two groups at 52 weeks after surgery and determine whether *E. coli*-derived rhBMP-2 with HA is non-inferior to auto-iliac bone graft in TLIF. According to a previous report, the interbody bone union rate was 92.7% on CT at 12 months postoperatively [14]. For the primary outcome analysis, we calculated that a sample size of 154 participants would provide at least 90% power to show the non-inferiority of rhBMP-2 with HA graft to autogenous iliac bone graft with a one-sided alpha value of 0.05 and a non-inferiority margin of 10% for the 12-month fusion rate of TLIF with the PEEK cage, assuming a 15% dropout rate at 12 months. The sample size was calculated by Power Analysis and Sample Size software, version 15 (NCSS, Kaysville, UT, USA). Therefore, this trial will recruit 154 participants to confirm the equivalence between the rhBMP-2 active group and auto-iliac bone graft control group in TLIF.

### Safety reporting

Safety will be assessed by evaluating all adverse events and effects which is related to surgery. If unexpected severe side effect occurs during the study, the researcher will report them to the IRB of the hospital and research staff as soon as possible and follow up continuously until the complications disappear or stabilize.

### Statistical analysis

Modified intention-to-treat (mITT) and per-protocol (PP) analyses will be conducted. The primary outcome will be analyzed using the PP strategy. The interbody fusion rate at 12 months after surgery in the rhBMP-2 group will be considered non-inferior to that in the auto-iliac graft group if the lower limit of the 95% confidence section of the fusion rate in the rhBMP-2 group is limited to within the non-inferiority margin of 10%. The mITT strategy will be the principle analysis for secondary outcomes, and we will analyze whether participants received a randomized graft material to reduce the effects of crossover and drop out that could disable the randomization of both groups assigned.

To evaluate the randomized graft material effect on clinical outcomes (i.e., ODI, EQ-5D-5L, and VAS pain scores), a linear repeated-measures mixture model is going to be used. Other radiographic and clinical outcomes and side effects between the two groups will be analyzed used by the chi-square test for categorical variables and the Student’s t test for continuous variables.

All statistical analyses will be performed using Stata/MP 17.0 (StataCorp LLC, College Station, TX). Statistical significance will be defined as a two-sided *p* value < 0.05, except for the *p* value from the non-inferiority test, which was one-sided.

## Discussion

This study is designed to confirm the effectiveness and safety of *E. coli*-derived rhBMP-2 in one- or two-level TLIF. To analyze the efficacy of *E. coli*-derived rhBMP-2, the primary outcome, the 12-month interbody fusion rate on CT after surgery, will be compared to that of auto-iliac bone graft. We will also analyze clinical outcomes, such as ODI, EQ-5D-5L value, and VAS scores, radiographic outcomes, and complications.

Several studies have been conducted on the efficacy of rhBMP-2 in lumbar fusion surgery, and the fusion rate has been found to be equal to or higher than that of auto-iliac bone graft [6, 15, 16]. In addition, the use of rhBMP-2 has the advantage in that no morbidity occurs unlike when auto-iliac bone graft is harvested [17]. Therefore, rhBMP-2 has emerged as a good substitute for auto-iliac bone graft in spinal fusion surgeries. However, the use of rhBMP-2 in fusion surgery has side effects such as heterotrophic ossification, radiculitis, and end plate changes [14, 18–20]. In spite of the dosage-dependent side effects, rhBMP-2 has been widely utilized off-label without a defined dose. In the published literature, doses varied considerably from 40 mg in the AMPLIFY study to less than 1-mg per level, with a mean dose/level of 4 mg [15]. Fortunately, earlier research failed to detect a connection between rhBMP-2 dose and fusion rate [15]. In a retrospective case series, Lytle et al. reported that the use of 1 to 2 mg/level rhBMP-2 improved the fusion rate, although greater doses had no effect [21]. In the other known investigations, rhBMP-2 doses reported in prior research did not increase fusion rates [15]. Due to a lack of evidence for dosages smaller than 1.05 mg/level, we are not able to make definitive conclusions other than that there is no increased benefit for fusion by increasing the rhBMP-2 dose higher than 1.05 mg/level, which is the smallest commercially available dose. In addition, although minimal-dose studies for posterolateral fusion have been conducted, studies on TLIF are limited [22]. To confirm this, we will conduct a study using a low dose (0.5–1 mg per segment) of *E. coli*-derived rhBMP-2 to confirm the safety threshold of *E. coli*-derived rhBMP-2 and its efficacy.

Similar to other growth factors, rhBMP-2 requires a carrier system capable of facilitating optimal cellular and vascular development, cellular attachment, and release kinetics [23]. Highly soluble, rhBMP-2 necessitates solid scaffolds that serve as drug carriers at the bone graft site for extended periods of time [24]. Ideal scaffolds should restrict growth factor release and avoid breakdown. Numerous materials have been proposed; however, absorbable collagen sponge has been the most extensively studied carrier for CHO-rhBMP-2 due to its strong binding and retention capacities. According to previous study, the combination of rhBMP-2 with collagen sponge may result in faster bone growth in postoperative bone deficiencies compared to standard bone grafting [15]. In bone graft site, collagen lacks both osteoconductivity and mechanical stability. Due to their space-providing qualities, calcium phosphates such as HA and beta-tricalcium phosphate have been regarded ideal candidates for a *E. coli-derived* rhBMP-2 delivery system [25]. Enhancing the osteoinductive action of *E. coli*-derived rhBMP-2 requires stable carriers with strong osteoconductive activity and high affinity for rhBMP-2. HA granules were still visible on CT images six months postoperatively, indicating HA's durability as a carrier [25]. Several investigations have suggested that its affinity with *E. coli*-derived rhBMP-2 is greater [25–27]. In these experiments, the usefulness of HA as a carrier for *E. coli*-derived rhBMP-2 was also verified. In our investigation, *E. coli*-derived rhBMP-2 will be combined with HA to investigate whether HA is acceptable for usage in conjunction with *E. coli*-derived rhBMP-2 during TLIF surgery.

Chinese hamster ovary (CHO) rhBMP-2 has a disadvantage in that the extraction efficiency is low in terms of cost*. E. coli*-derived rhBMP-2 is economical because it is cheaper than CHO-rhBMP-2 and can be produced in larger quantities [28]. In addition, bone induction capacity was similar compared with CHO-rhBMP-2 in an in vivo study [29]. There is no RCT on *E. coli*-derived rhBMP-2, except for its use in PLF in spinal surgery. Therefore, we aim to examine the effect of *E. coli*-derived rhBMP-2 in TLIF.

This study will be the first of its kind to analyze the effectiveness and safety of *E. coli*-derived rhBMP-2 with HA in one- to two-segment TLIF surgery. This study is designed to evaluate the non-inferior results between *E. coli*-derived rhBMP-2 with HA and auto-iliac bone graft using an appropriate sample size, blinded analyses, and prospective registration to reduce bias. This trial result will provide high-level evidence of the efficacy, safety, and standard indicators for the use of *E. coli*-derived rhBMP-2 in TLIF surgery.

## Data Availability

The electronic database server will not be publicly accessible. Access to the data set is provided only to the Data Management Committee of our research group. The study findings will be published in a peer-reviewed journal.

## Abbreviations

rhBMP-2: Recombinant human bone morphogenetic protein-2
LLIF: Lateral lumbar interbody fusion
ALIF: Anterior lumbar interbody fusion
RCT: Randomized controlled trial
HA: Hydroxyapatite
IRB: Institutional review board
VAS: Visual analog scale
PEEK: Polyetheretherketone
CT: Computed tomography
mITT: Modified intention-to-treat
PP: Per-protocol
PLF: Posterolateral fusion
ODI: Oswestry Disability Index
EQ-5D-5L: EuroQol-5-dimensions-5-level

## Declarations

### Current stage of study

Some hospitals are conducting trials by recruiting participants who meet the inclusion criteria, obtaining explanations and consent.

### Related articles

There are no publications containing the results of this study.
